# Supplementary material for: Mesenchymal Stem/Stromal Cells Derived from Cervical Cancer Promote M2 Macrophage Polarization
Source: Cells. 2023 Mar 30;12(7):1047. doi: 10.3390/cells12071047 (PMC10093665; doi:10.3390/cells12071047)
Supplement: Supplementary file 1 [file cells-12-01047-s001.zip › cells-2182515-supplementary.pdf]

Supplementary Materials

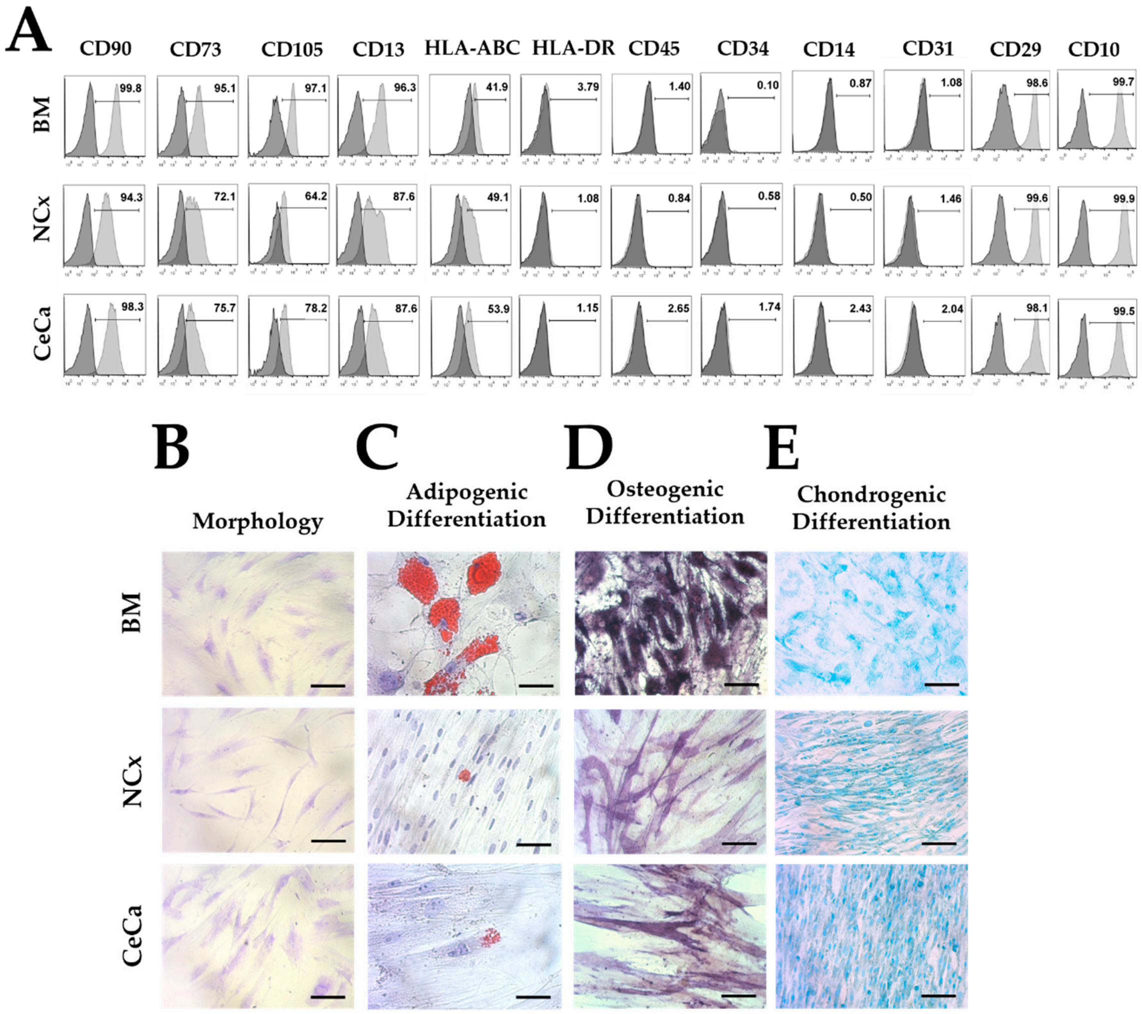

Supplementary Figure S1: Characterization of BM-MSCs, CeCa-MSCs and NCx-MSCs. A) Histograms representative of the evaluation of characteristic markers of MSCs at 4 and 5 passage; positive for CD90, CD73, CD105, CD13, HLA-ABC, CD29, CD10 and negative for HLA-DR, CD45, CD34, CD14 and CD31. **B)** Morphology, observed with Wright stain (bar size 100 microns). **C)** Adipogenic differentiation capacity, observed with oil red O (bar size 200 microns). **D)** Osteogenic differentiation capacity, observed via alkaline phosphatase reaction (bar size 100 microns). **E)** Chondrogenic differentiation capacity, observed with alcian blue (bar size 100 microns). Six samples from each source were analyzed. A representative result is showed.

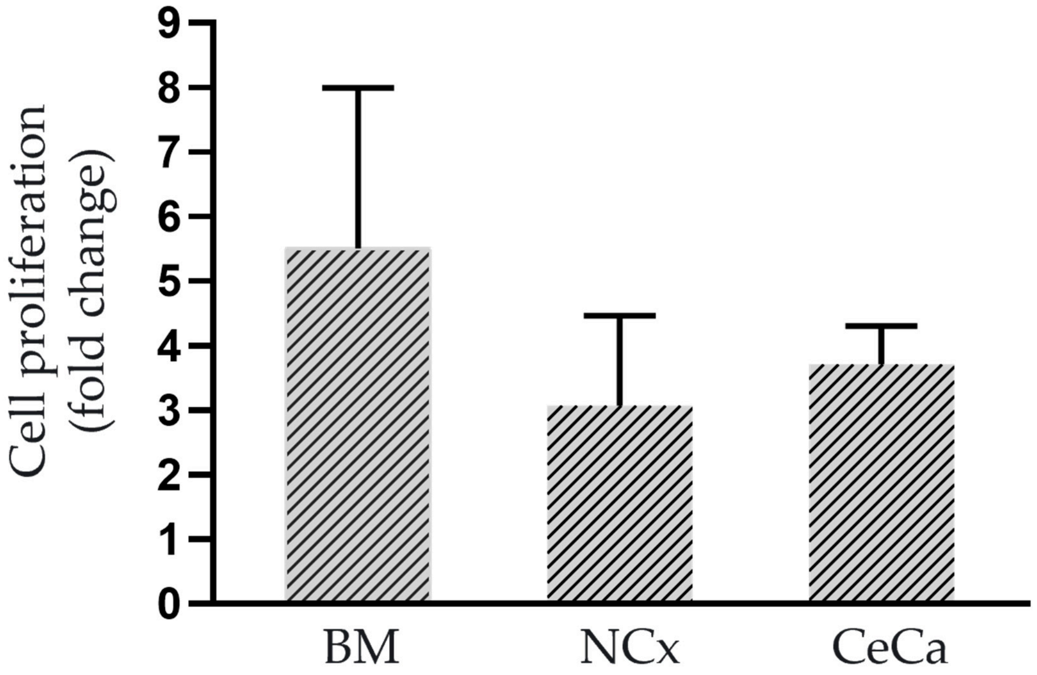

Supplementary Figure S2: Similar proliferation potential of MSCs from BM, NCx and CeCa. 100,000 MSCs at passage 4 from each source were cultured for 7 days and cell proliferation (fold change) was measured after culture. MSCs were harvested with trypsin and counted using trypan blue staining; the viability percentage in all cultures was greater than 95%. Bar graphs represent the mean and standard deviation from six individual experiments (n=6), from each analyzed source.

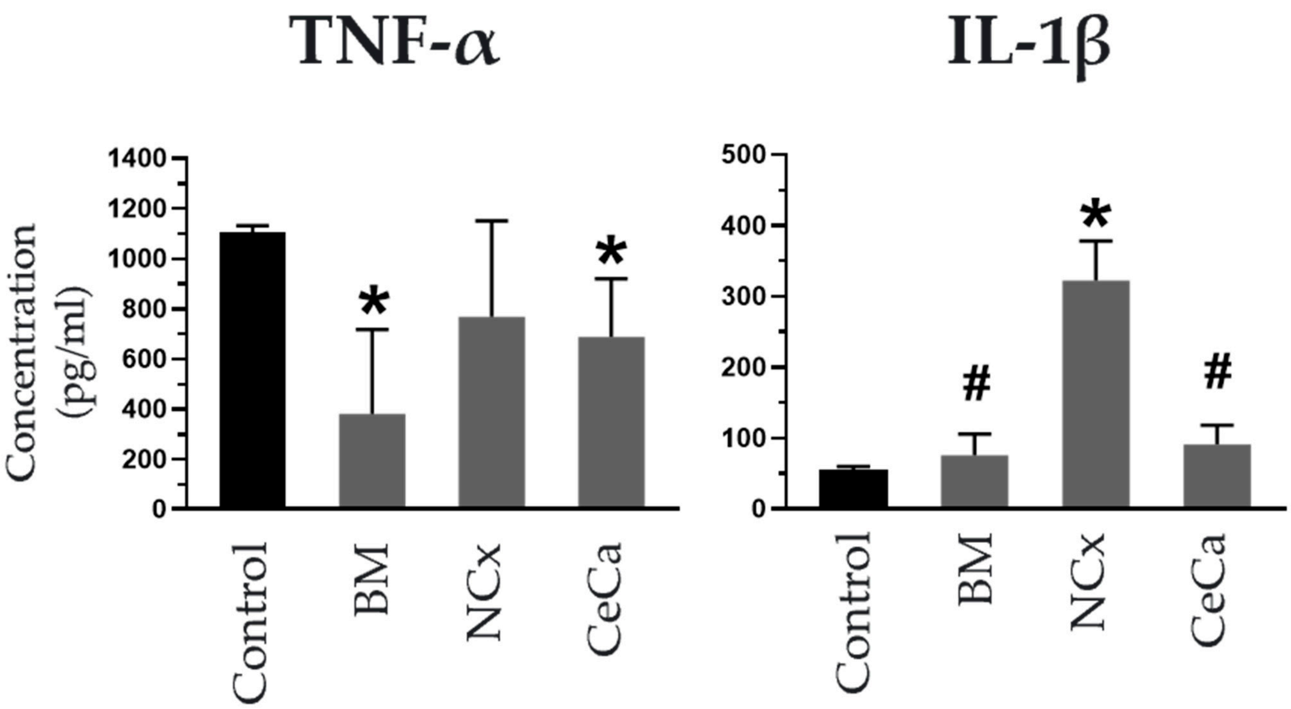

Supplementary Figure S3: The presence of NCx-MSCs, unlike CeCa-MSCs, increases the soluble concentration of inflammatory molecules in a macrophage coculture system with M1 inducer media. Bar graphs represent the mean and standard deviation. *significant difference from the control, # significant difference from NCx-MSCs ($p < 0.05$); $n = 6$.
